# Landscape analysis of available European data sources amenable for machine learning and recommendations on usability for rare diseases screening

**DOI:** 10.1186/s13023-024-03162-5

**Published:** 2024-04-06

**Authors:** Ralitsa Raycheva, Kostadin Kostadinov, Elena Mitova, Georgi Iskrov, Georgi Stefanov, Merja Vakevainen, Kaisa Elomaa, Yuen-Sum Man, Edith Gross, Jana Zschüntzsch, Richard Röttger, Rumen Stefanov

**Affiliations:** 1 Department of Social Medicine and Public Health, Faculty of Public Health, Medical University of Plovdiv, Plovdiv, Bulgaria; 2 Bulgarian Association for Promotion of Education and Science, Institute for Rare Disease, Plovdiv, Bulgaria; 3 Pfizer Biopharmaceuticals Group, Medical Affairs, Helsinki, Finland; 4 Takeda Oy, Helsinki, Finland; 5 Global Medical Affairs Rare Disease, Novo Nordisk Health Care AG, Zurich, Switzerland; 6 EURORDIS – Rare Diseases Europe, 96 Rue Didot, Paris, 75014 France; 7 Department of Neurology, University Medical Center, Göttingen, Germany; 8 Department of Mathematics and Computer Science, University of Southern Denmark, Odense, Denmark

**Keywords:** Databases, Health data, Electronic health records, ERNs, Rare diseases, Machine learning (ML), Artificial intelligence (AI), FAIR, Legislation, Consent

## Abstract

**Background:**

Patient registries and databases are essential tools for advancing clinical research in the area of rare diseases, as well as for enhancing patient care and healthcare planning. The primary aim of this study is a landscape analysis of available European data sources amenable to machine learning (ML) and their usability for Rare Diseases screening, in terms of findable, accessible, interoperable, reusable(FAIR), legal, and business considerations. Second, recommendations will be proposed to provide a better understanding of the health data ecosystem.

**Methods:**

In the period of March 2022 to December 2022, a cross-sectional study using a semi-structured questionnaire was conducted among potential respondents, identified as main contact person of a health-related databases. The design of the self-completed questionnaire survey instrument was based on information drawn from relevant scientific publications, quantitative and qualitative research, and scoping review on challenges in mapping European rare disease (RD) databases. To determine database characteristics associated with the adherence to the FAIR principles, legal and business aspects of database management Bayesian models were fitted.

**Results:**

In total, 330 unique replies were processed and analyzed, reflecting the same number of distinct databases (no duplicates included). In terms of geographical scope, we observed 24.2% (*n* = 80) national, 10.0% (*n* = 33) regional, 8.8% (*n* = 29) European, and 5.5% (*n* = 18) international registries coordinated in Europe. Over 80.0% (*n* = 269) of the databases were still active, with approximately 60.0% (*n* = 191) established after the year 2000 and 71.0% last collected new data in 2022. Regarding their geographical scope, European registries were associated with the highest overall FAIR adherence, while registries with regional and “other” geographical scope were ranked at the bottom of the list with the lowest proportion. Responders’ willingness to share data as a contribution to the goals of the Screen4Care project was evaluated at the end of the survey. This question was completed by 108 respondents; however, only 18 of them (16.7%) expressed a direct willingness to contribute to the project by sharing their databases. Among them, an equal split between pro-bono and paid services was observed.

**Conclusions:**

The most important results of our study demonstrate not enough sufficient FAIR principles adherence and low willingness of the EU health databases to share patient information, combined with some legislation incapacities, resulting in barriers to the secondary use of data.

**Supplementary Information:**

The online version contains supplementary material available at 10.1186/s13023-024-03162-5.

## Background

In the European Union (EU), a rare disease (RD) is one that affects no more than 1 person per 2,000 population. There are between 6,000 and 8,000 different RDs that afflict an estimated 30 million citizens in Europe [1].

Although RDs are highly diverse in terms of etiology, pathophysiology, and clinical manifestation, they have common characteristics: many RDs are severe, chronic, and life-threatening and finding the proper diagnosis presents a significant barrier in their treatment [2]. Affected people are frequently faced with multiple years of burdensome diagnostic journey with misdiagnoses, and an average diagnosis delay of up to 8 years [3]. Furthermore, there are no approved therapies for nearly 90% of these disorders [4]. RDs are now more widely acknowledged as a serious public health issue that affects people all over the world and places an undue financial burden on patients, families, and healthcare systems [5].

Effective methods for improving medical care for RD patients include projects and networks that aim to aggregate information and expertise so that healthcare professionals can easily access and share essential data [6]. One of the most comprehensive knowledge bases for RDs is Orphanet [7], which provides information on RDs, and links to specialist centers, patient organizations, and other resources. Other European initiatives include the European Reference Networks (ERNs), the European Joint Program on Rare Diseases (EJP RD), and RDConnect [8].

Patient registries and databases are essential tools for advancing clinical research in RDs, as well as for enhancing patient care and healthcare planning. They are the only means of data pooling that can result in an adequate sample size for epidemiological and/or clinical research [9].

Hundreds of registries and other databases at the national, regional, and local levels^1^ in Europe gather information about RD patients. As this information is widely spread, an important tool for researchers, medical professionals, patients, and policymakers is the EU Rare Disease Platform. It aims to provide its users with a standardized instrument to improve knowledge, diagnosis, and treatment of RDs while harmonizing data collection and interchange at the EU level [10].

The design, development, and establishment of a registry, or another type of patient database, involves different factors, including the technicalities of coding languages and data-capture programs; ethical and legal concerns to ensure data privacy and protection while also enabling data sharing and reuse; governance and considerations regarding the various interests of patients, clinicians, researchers, policymakers, and other stakeholders. RD registries are being supported through worldwide initiatives, such as EPIRARE (European Platform for Rare Disease Registries) which seeks to address the regulatory, legal, ethical, and technical difficulties in the registration of European RD patients [11].

In addition to these collaborative efforts and global platforms, advancements in information technology (IT), particularly in the fields of artificial intelligence (AI), are important elements that can enhance the situation of patients with RDs. It is essential to increase the accessibility of data sources, including hospital information systems (HISs), electronic health records (EHRs), and health-related registries, to develop systems that could assist clinicians in their diagnostic decisions. The value of sophisticated analysis techniques like machine learning (ML) in clinical decision-making has been proven by a review of AI-based clinical decision-support technologies [12]. The usage of data sources based in Europe is closely related to legal and ethical standards within the European legislative framework, and it also needs to be facilitated through the FAIR principles for data management (Findability, Accessibility, Interoperability, and Reusability).

A recent review evaluated European RD databases in terms of fulfilment of FAIR principles and meeting EU regulation challenges while considering their potential for genetic newborn screening using AI-based tools [13]. As RDs primarily affect children and account for about 80% of all cases being of genetic origin [14], genetic screening for early RDs identification is of growing importance. The review examined key organizational, FAIR and legal challenges identified during European RD databases mapping which may impede the implementation of ML-based screening technologies for RD patients. It was elaborated within the frame of the European project Screen for Care (Screen4Care), a project aiming to shorten the path to RD diagnosis by using newborn genetic screening and digital technologies [15]. Screen4Care is focusing on the early detection of RDs via advanced IT and clinical decision support tools, using AI and ML. It includes the development of a federated metadata repository amendable to federated ML algorithms,^2^ based on existing RD databases. Regarding RD database management, challenges identified include the need for better data quality, sustainability, funding, and governance of RD registries; establishing FAIR-compliant databases and considering the necessity to adapt the legal framework for reliable data collection and accelerated interoperability across Europe, offering further opportunities for RD patients [13].

## Materials and methods

### Aim

The primary aim of this study is a landscape analysis of available European-wide data sources amenable for ML and their usability for Rare Diseases screening, in terms of FAIR, legal, and business considerations. Second, recommendations will be proposed to provide a better understanding of the health data ecosystem – accessibility, sharing, interoperability, legislation, etc. – to inform Screen4Care Project tasks of further steps.

### Design of the study and participants’ profile

In the period of March 2022 to December 2022, a cross-sectional study using a semi-structured questionnaire was conducted in accordance with the Checklist for Reporting Results of Internet E-Surveys (CHERRIES) [16, 17] (Additional file 2: Table 1. Checklist for Reporting Results of Internet E-Surveys (CHERRIES)). A non-random convenience sampling method was used in recruiting the participants and a list of potential respondents was prepared, including all individuals that had been identified as eligible to answer the questions – the main contact person of a database that could be: health-related registry, EMR, EHR, HIS, and repositories for genomics. Participants were recruited by using individual emails. In addition, based on the heterogeneity of the questionary a non-random snowball sampling method was applied to target experts with extensive experience in FAIR principles for database management, organization, level of access and metadata or broad knowledge about legal, ethical, and business practices in data collection and operation with a focus on consent and data ownership, sensitive information, data protection, legislation, data sharing and fees.

**Table 1:** FAIR components ranking by the posterior estimates of the Bayesian logit model according to database type

	**Findability**	**Accessibility**	**Interoperability**	**Reusability**	**Overall rank**
Genetic data	2	1	5	1	1
HIS	4	4	2	2	3
EMR	3	5	3	3	4
Registries	1	2	4	4	2
EHR	5	3	1	5	4

### Settings

Screen4Care is a European research project, run by an international public–private consortium of 35 partners. The project is funded by the Innovative Medicines Initiative (IMI 2JU), a joint undertaking of the European Union and the European Federation of Pharmaceutical Industries and Associations (EFPIA) and thus the geographical scope of our study aims to databases operating in EU and EEA countries.

### Eligibility criteria

EU and EEA health-related databases as registries, EMR, EHR, HIS, and repositories for genomics that include information about clinical data; laboratory tests; neurological assessment, or other specialized investigation; medical history, including premature rupture of membranes (PROM – Premature rupture of membranes); imaging studies: X-ray, MRI; diagnosis/confirmed diagnosis (e.g. ICD codes); operations/other interventions; medications/therapy; devices/type and collected parameter; health services; genetic data (including human phenotype ontology—HPO); and administrative and billing data.

### On-line questionnaire

The design of the self-completed questionnaire survey instrument was based on information drawn from the scoping review on challenges in mapping European RD databases, relevant to ML-based screening technologies in terms of organizational, FAIR and legal principles [13], based on relevant scientific publications, including both quantitative and qualitative research The survey contained 81 questions distributed over six main panels: 1) introduction; 2) administrative; 3) screening; 4) FAIR-ness; 5) legal and business; and 6) end of the survey. Question types were closed-ended single-choice questions, semi-closed selective questions with a text answer, semi-closed enumerated questions with/without a text answer, a matrix of questions and open-ended questions. Detailed information about the questionnaire’s structure and content is available in Additional file 3: Table 1. Structure and content of the questionnaire survey instrument.

An electronic questionnaire form on a landscape analysis of available data sources and their usability of RDs screening in Europe was developed using the LimeSurvey platform Enterprise plan version and was distributed to 3032 potential respondents. The questionnaire introductory panel started with a general description of the Screen4Care project, outlined the aim of collecting particular information on the topic of interest and ended with a consent statement for the use of the anonymous data, which was agreed to before filling out the survey questions by the respondent. The survey participation was entirely voluntary. In addition, the anonymous nature of the survey did not require ethics committee approval. The study was conducted according to ethical guidelines established by the Declaration of Helsinki [18].

### Statistical analysis

Descriptive statistics were used to present a univariate analysis of the data. Discrete variables were presented with absolute numbers and proportions, whereas median and 25^th^ and 75^th^ percentiles were used for continuous ones. To determine database characteristics associated with the adherence to the FAIR principles, legal and business aspects of database management Bayesian models were fitted. Positive response for the outcomes of interest was used as a dependent variable in the models. Non-informative priors from the binomial distributions were applied. The outcomes were measured by “yes” and “no” coding to predefined questions of interest. Then the total number of responders for each defined question was used for proportion estimation. The models assumed a logit link function and were fitted using the rstanarm package [19] with four Monte-Carlo chains stimulation for each 2000 iteration per model fit. Convergence was assessed using the Gelman-Rubin diagnostic [20] and the effective sample size (ESS) was calculated for each parameter. The results were presented as posterior median and 95% credible intervals (95% CI). The models were fitted using the R software version 4.3.1 [21].

## Results

### Databases’ profile

In total, 330 unique replies were processed and analyzed, reflecting the same number of distinct databases (no duplicates included). In terms of geographical scope, we observed 24.2% (*n* = 80) national, 10.0% (*n* = 33) regional, 8.8% (*n* = 29) European,^3^ and 5.5% (*n* = 18) international^4^ registries coordinated in Europe. Over 80.0% (*n* = 269) of the databases were still active, with approximately 60.0% (*n* = 191) established after the year 2000 and 71.0% last collected new data in 2022. The frequency with which a database was updated varied extensively: 15.5% (*n* = 51) of respondents perform the action once per month, 6.4% (*n* = 21) once every six months, 5.8% (*n* = 19) once a year, 22.4% (74) renew information using another interval approach, 2.7% (*n* = 9) could not provide an answer, and 47.3% (*n* = 156) did not respond. The median number of new cases introduced in the databases over the previous year of observation was 110 (31; 400) by 38.5% (*n* = 127) of the respondents. The median number of observations / cases in the databases (32.1%, *n* = 106) was calculated to be 966 (300, 6888). The median number of active cases / patients included in the databases (43.6%, *n* = 144) was reported to be 1,400 (251; 5,893). In 33.0% (*n* = 112) of databases, information concerning the patient's death was collected.

**Fig. 1:**
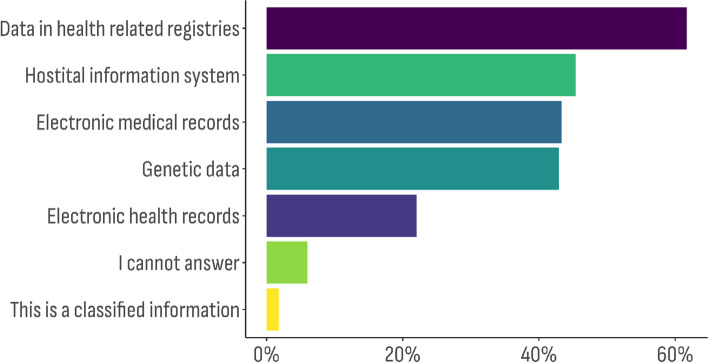
Distribution of the databases characteristic by type of data the organization is operating with (the overall percentage exceeds 100%, because this was a semi-closed enumerated question)

**Fig. 2:**
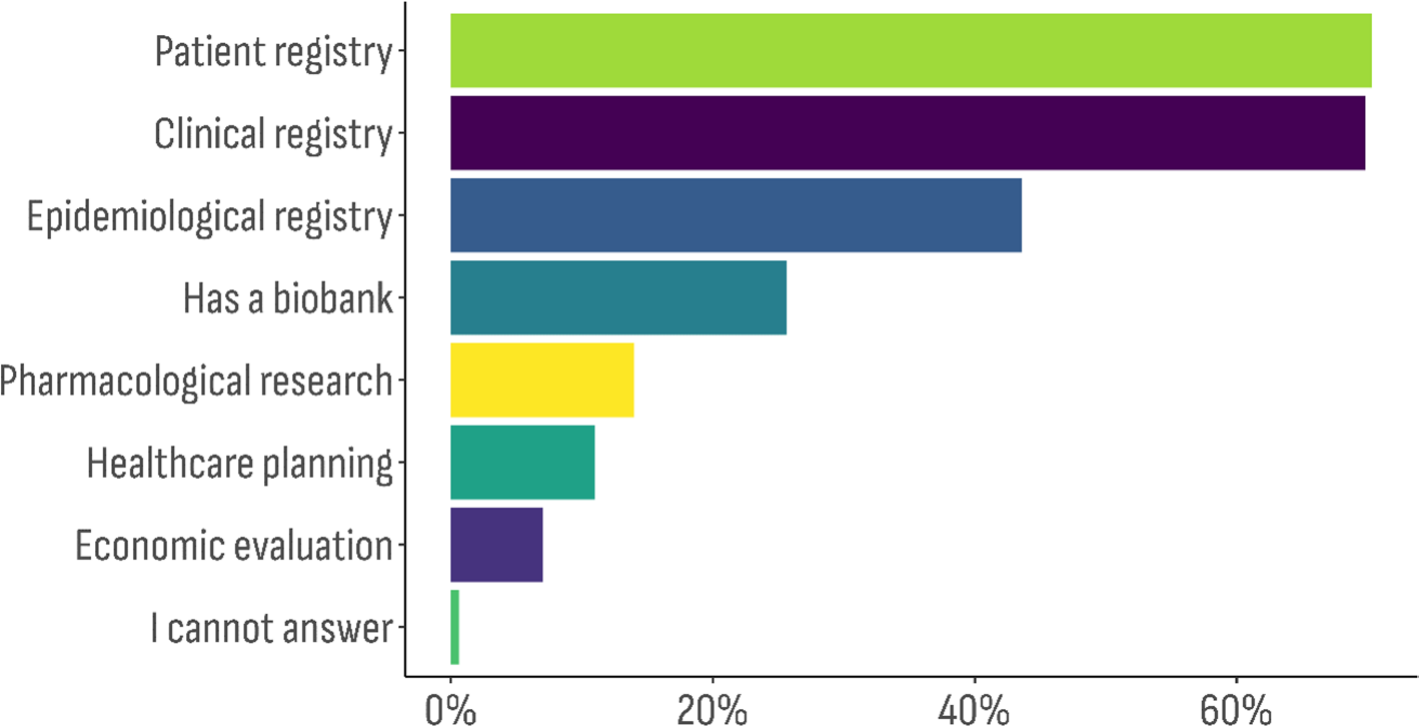
Distribution of the databases characteristic by type of the registry (the overall percentage exceeds 100%, because this was a semi-closed enumerated question)

**Fig. 3:**
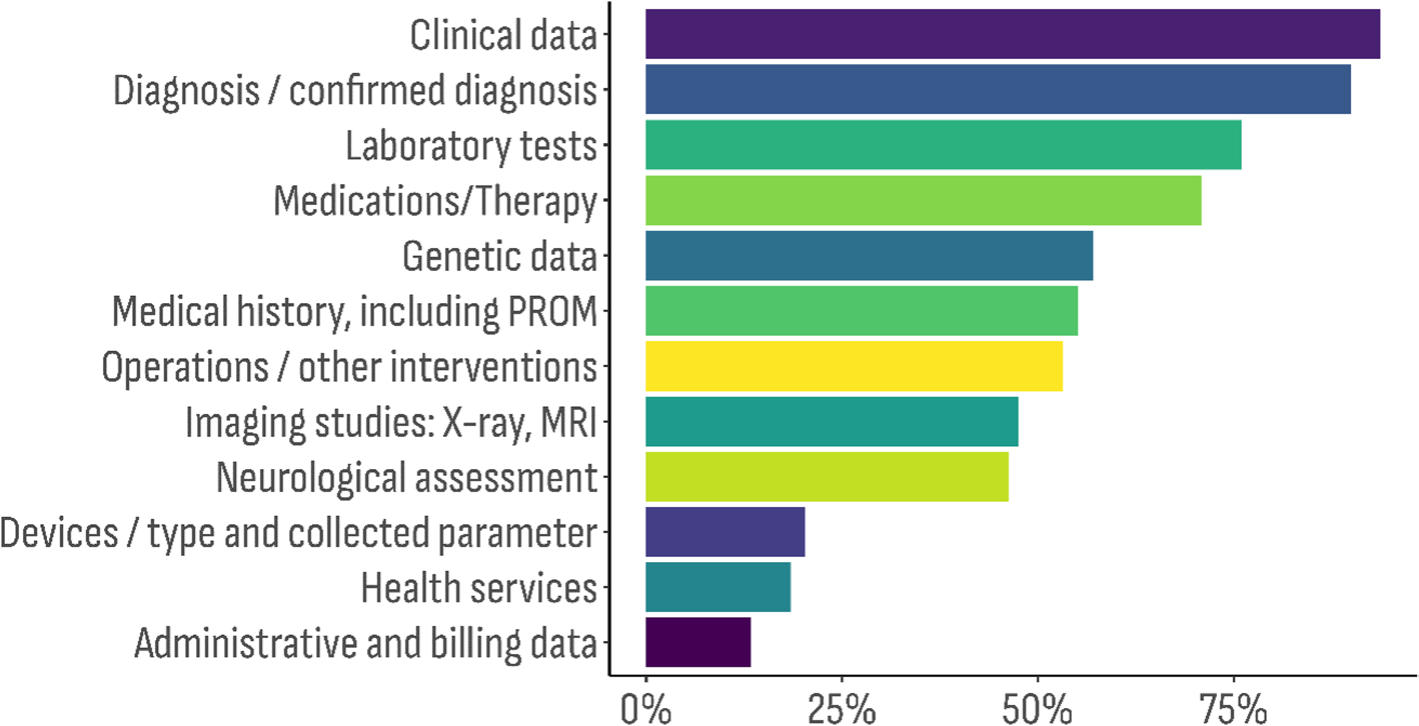
Distribution of the databases characteristic by type of data categories included (the overall percentage exceeds 100%, because this was a semi-closed enumerated question)

The multiple response sets (semi-closed enumerated questions) provided the following information – 1) Databases’ operational data (*n* = 629) (Fig. 1); 2) Registry types (*n* = 416) (Fig. 2); and 3) Type of data categories (*n* = 1013) included (Fig. 3).

Other sets of multiple response questions were focused on collecting information about the database characteristics relevant to RDs. We aimed to identify the RDs groups (*n* = 446) the databases contained information about – metabolic and endocrine disorders: 30.0% (*n* = 86); neurological and neuromuscular disorders: 29.3% (*n* = 84); hematological diseases: 17.4% (*n* = 50); beta oxidation disorders: 9.4% (*n* = 27); other rare conditions: 61.7% (*n* = 177); I cannot answer: 7.7% (*n* = 22). More detailed information about the distribution of subgroup disorders is available in Additional file 1: Table 1. Main rare disease groups and subgroup distribution.

### FAIR-ness of the organization

The FAIR section of the survey aimed at evaluating the degree of adherence of the responder’s databases to the pre-defined FAIR principles. The section was optional for respondents to complete based on their level of expertise. The section was structured around the 15 principles, serving as guidelines to improve the accessibility, interoperability, and reusability of digital resources [22]. Each FAIR principle was scrutinized by the S4C consortium based on existing evidence [23] and the S4C objectives [15]. The aggregated results were summarized using counts and percentages in Additional file 1. While each principle includes several specific criteria, the evaluation of the organization's FAIR compliance focused on only five questions chosen for their alignment with the core concept and their relevance to the further development of machine learning solutions in the field of rare diseases. The overall FAIR-ness of the database was assessed by the question “Are the FAIR principles explicitly mentioned in the database policy of your organization?”. A positive outcome would be considered if the responder had answered, “All FAIR principles are explicitly mentioned”.

The principle of “findability” stresses the importance of unique identifiers and detailed metadata for digital resources [23]. Adherence to this principle requires registering resources in searchable repositories. In our analysis, the concept of “findability” was evaluated based on a positive answer to the questions “Are the data produced and/or used in the database discoverable with metadata, identifiable and locatable by means of a standard identification mechanism (e.g., persistent and unique identifiers such as Digital Object Identifiers)?”. The “accessibility” principle emphases on open, universally implementable access protocols and authentication procedures. It also underscores the importance of persistent metadata availability even if data is unavailable. Implementation of this principle involves defining machine-actionable metadata persistence templates [23]. In the context of rare diseases, ensuring actual access to datasets for machine learning was a specific concern. Therefore in our evaluation, the “accessibility” dimension was based on positive affirmations that “all datasets can be accessed and made available” in response to the question “Which data produced and/or used in the database could be made available?”.

The principle of “interoperability” aims to facilitate understanding and interoperability between different resources, especially in interdisciplinary studies. It also promotes the use of structured sets of terms to avoid ambiguity and highlights the importance of including qualified references to other data or metadata [23]. Several vocabularies and ontologies are available to facilitate the implementation of this principle. However, the FHIR (Fast Healthcare Interoperability Resources) standard was addressed as a potential candidate for data in the field of rare diseases [15]. Therefore the “interoperability” principle was evaluated by positive answer to the question “Does your organization (data site) follow the FHIR standard to support eSource data exchange”.

Finally, the “reusability” principle underscores the significance of pertinent attributes to describe digital resources, thereby amplifying their potential for reuse. Its goal is to empower users to evaluate the appropriateness of identified resources for particular tasks [23]. Clear licensing terms are crucial for mitigating legal ambiguities and promoting extensive reuse. This aspect of the principle was determined to be critical for S4C objectives and therefore was evaluated by a positive answer to the question, “Are the data licensed to permit the re-use possible?” Out of a total of 330 survey respondents, 141 (42.7%) participated in this section. The distribution of responses is available in Additional file 1: Table 2. FAIR database characteristics. Regarding overall adherence to FAIR principles, a total of 25 (17.9%) reported full compliance with all 4 components, while more than half either could not provide an answer (*n* = 56, 40%) or indicated that the FAIR principles were not being applied on the site (*n* = 23, 16.4%). Only 2 (1.43%) revealed partial database compliance, specifically in terms of findability and accessibility.

Explored systematically by all FAIR components, the majority of respondents (*n* = 31, 51.7%) indicated that the data repository had a unique and persistent identifier (PID). Moreover, 14 (23.3%) identified several data releases with versions attached. Regarding data accessibility, almost half of the responders (*n* = 28; 45.9%) ensured accessibility either via a web browser or API. Full data access was reported in 15 (25.0%) of the databases. Interoperability of the data was indicated by 43 (71.7%) of the respondents, and four of them (6.8%) reported implementation of the observational medical outcome model partnership (OMOP). Data licensing enabling re-usability was applied in 20 (47.6%) of the responder’s databases.

**Fig. 4:**
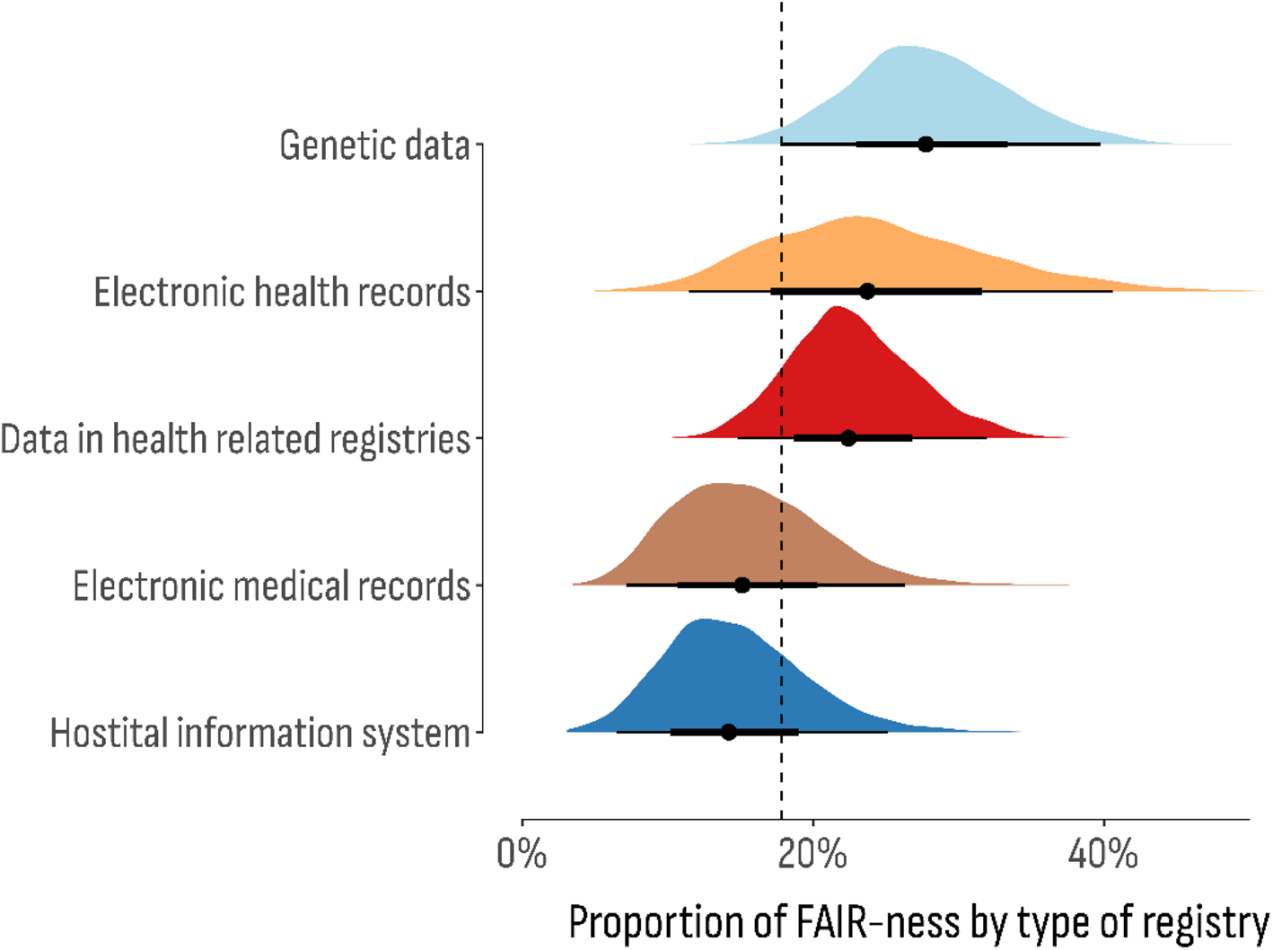
Posterior distribution of the estimated proportion of overall adherence to FAIR principles by the type of the databases

Posterior estimates from that Bayesian model (Fig. 4) showed that the proportion of positive responses to the overall FAIR-ness was highest for genetic databases (28.12%; 95% CI 17.7%–39.8%), followed by data in health-related registries (22.7%; 95% CI 14.8%–32.0%), EHR (24.4%; 95% CI 11.5%–40.6%), HIS (14.6%; 95% CI 6.5%–25.2%), and EMR (15.5%; 95% CI 7.2%–26.3%). In terms of database reusability, on average, 34% of all respondents shared a positive outcome in this fair dimension.

**Fig. 5:**
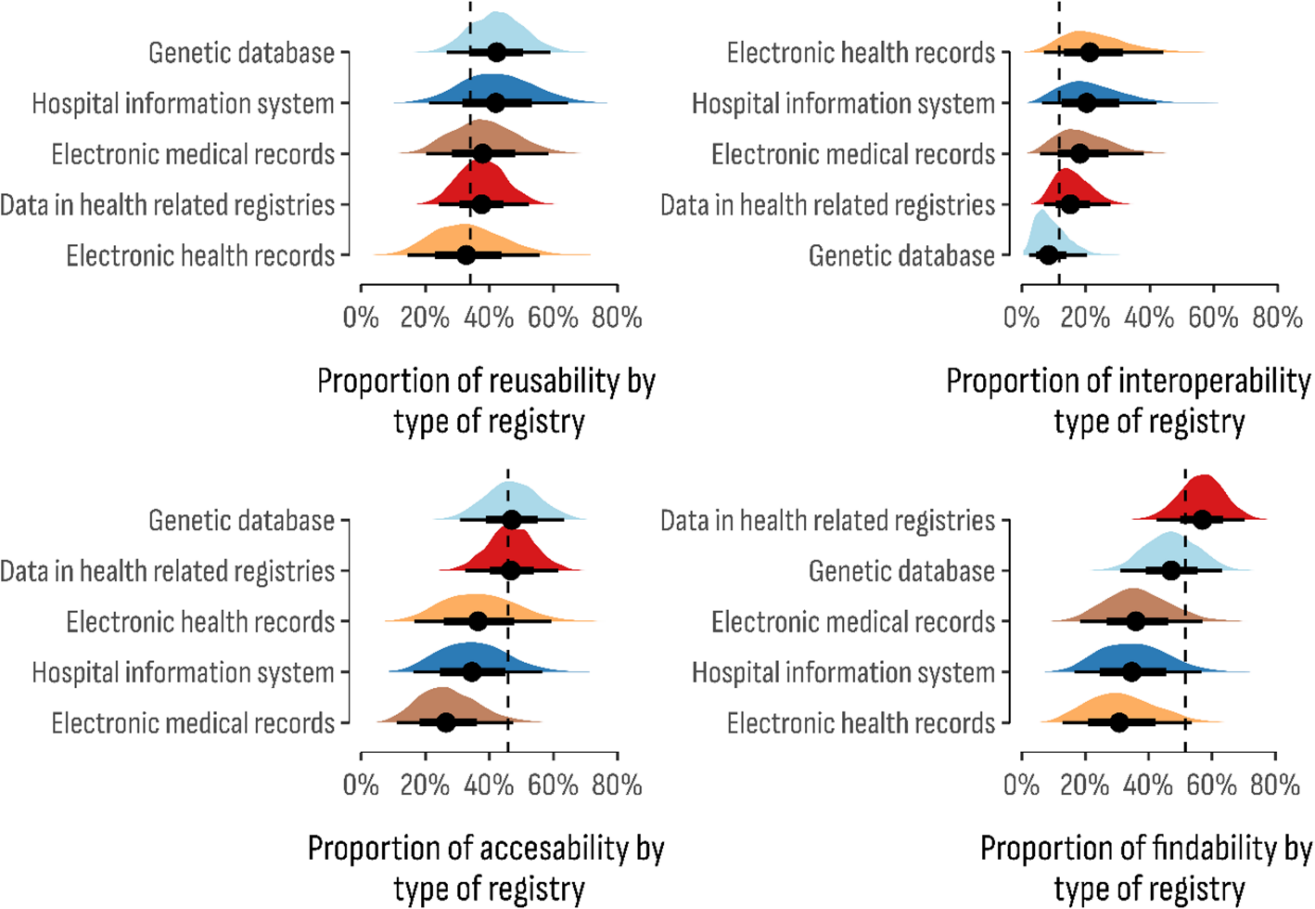
Posterior distribution of the estimated proportion of each component of the FAIR principles by the type of the databases

However, small discrepancies could be noticed according to the database type (Fig. 5). For instance, the positive reusability outcome was more frequent in hospital information systems (41.2%; 95% CI 21.2%–64.5%) and genetic databases (42.3%; 95% CI 26.5%–59.1%), while the least reusable were databases of electronic health records (32.3%; 95% CI 14.5%–55.7%). The highest proportion of interoperable databases was reported for electronic health records (21.3%; 95% CI 6.9%—44.2%) and hospital information systems (20.3%; 95% CI 6.3%-42.2%), while genetic data was estimated as the least interoperable (8.4%; 95% CI 2.2%-20.4%). However genetic databases were found with the highest level of accessibility (47.0%; 95% CI 30.7%–63.3%), while the lowest level was estimated in electronic health records (25.8%; 95% CI 11.5%–45.1%). Furthermore, genetic databases were also prominent in terms of their findable databases (53.8%; 95% CI 37.2%–69.8%), while the lowest estimated proportion was in electronic health records (36,4%; 95% CI 16.5%-59.2%).

Based on the posterior estimates, a ranking-based approach was used to determine the FAIR adherence order by the database type. As shown in Table 1, genetic databases were considered the most adherent to the evaluated FAIR criteria, while health-related registries and electronic health records were at the bottom of the ranking.

A similar approach was to evaluate the impact of the disease type reported in the dataset and the geographical scope for the responders of registry-type databases (Table 2). The highest overall FAIR adherence was listed for databases containing information for “other” RDs and neuromuscular disorders. Regarding their geographical scope, European registries were associated with the highest overall FAIR adherence, while registries with regional and “other” geographical scope were ranked at the bottom of the list with the lowest proportion.

**Table 2:** Overall FAIR adherence – ranking by the posterior estimates of the Bayesian logit model according to the type of diseases included in the database and geographic scope (only for registries)

Disease type	Median	2.5% CI	97.5% CI	Rank
Beta oxidation	6.2%	0.2%	27.9%	4
Endocrine	13.1%	4.8%	26.9%	3
Haematological	3.3%	0.1%	15.2%	5
Neuromuscular	18.8%	9.0%	32.7%	2
Other	22.1%	14.2%	31.6%	1
**Geographical scope**				
European registry	50.4%	27.4%	74.5%	1
International registries coordinated in Europe	20.3%	3.2%	54.2%	3
National registry	20.6%	10.5%	34.6%	2
Other	13.8%	0.7%	53.2%	4
Regional registry	3.6%	0.2%	17.1%	5

### Legal and business characteristics

The legal and business section included questions, focused on the management of health data which responders’ organization collects and operates with. Six domains of interest were identified: consent and data ownership; sensitive information; data protection; legislation; data sharing and fees.

The consent and data ownership domain were evaluated by 3 questions in the survey. The first outcome for this domain was a positive answer to the question “Do you require re-consent of patients when data is used in ways that do not fall within the original purpose of the registry?” As a second outcome, positive answers (all types of consent) to the question “What type of consent do you collect?” were used. Finally, the third outcome was a negative answer (No consent) to the question “Which of the following consent models do you apply for sharing anonymized patient health information in network electronic exchange for research purposes?”.

Data ownership was evaluated by the question, “Are patients aware that their information may be used for further research, monitoring performance, service planning, auditing, quality assurance purposes, etc.?” and the outcome for that domain was a negative response. Data protection was assessed by the question “Are there national health data security policies regarding the technical standards to be used to ensure health data for primary use are processed and stored securely?”. Responders who answered “There is one national data security policy” or “There are several national data security policies” were combined and used as a positive outcome for this domain. The legislation was evaluated by the estimated share of responders who answered “Yes” to the question “Are there legislative provisions concerning the primary and secondary use of data?”. The final domain was estimated by the proportion of respondents who were willing to share their databases as a contribution to the goals of the Screen4Care project.

The overall characteristics of database management were shown in Additional file 1: Table 3. Overall characteristics of database management – legal and business information. The vast majority of responders indicated that there was at least one national data security policy regulating the technical standards for data sharing (*n* = 84; 75.0%). Moreover, almost all respondents in this section shared that patients were aware that their information may be used for research, monitoring, service planning, and other data processes (*n* = 94; 83.0%). Less than a quarter of the databases did not require re-consent when data was used in ways that fell outside the original purpose of data collection (*n* = 25; 22.3%). Opt-in consent was the preferred model mode in the anonymized patient network-sharing process among participants (*n* = 18, 16.3%). In terms of the type of consent collected, most of the databases indicated that consent was collected for every use of data (*n* = 34; 30.4%). Genetics data as a sensitive data item was collected in 61 of the databases (48.4%), while mental health data was gathered in 19 (15.1%) of the responders’ databases. Ownership of the data (*n* = 101; 92.7%), along with data anonymization (*n* = 102; 93.6%), were observed as primary items for consideration in the data sharing process among participants. However, less noticeable items were “Salting of the database or the use of fake data to uncover unauthorized use and copying of the database” and “Fees to be charged and protection of the licensee from fee creep,” not considered in 38 (35.5%) and 34 (31.5%) of the database, respectively.

Responders’ willingness to share data as a contribution to the goals of the Screen4Care project was evaluated at the end of the survey. This question was completed by 108 respondents; however, only 18 of them (16.7%) expressed a direct willingness to contribute to the project by sharing their databases. Among them, an equal split between pro-bono and paid services was observed.

**Fig. 6:**
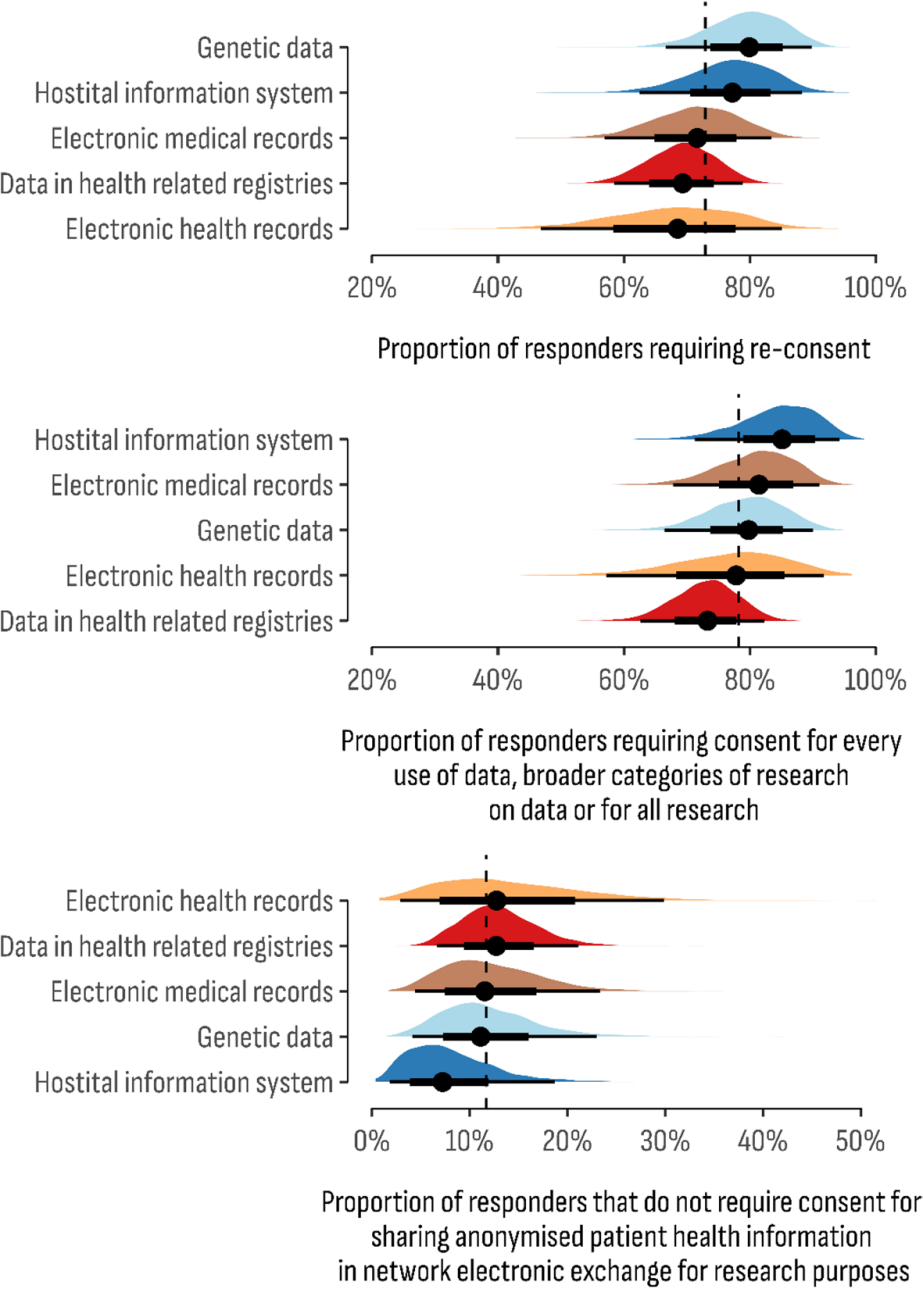
Posterior distribution of the estimated proportion of consent domain by the type of the databases

**Fig. 7:**
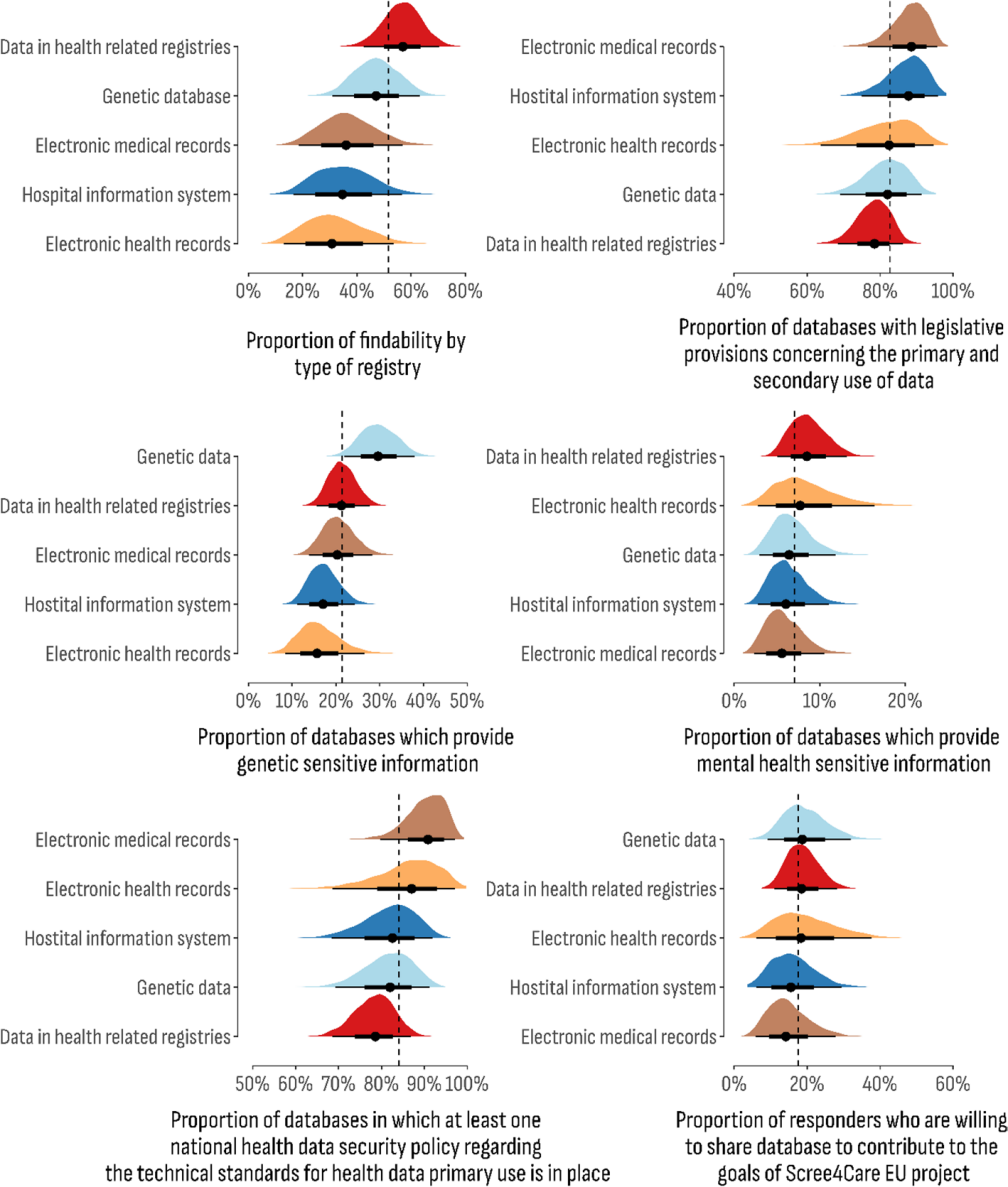
Posterior distribution of the estimated proportion of legal and business information items by the type of the databases

Posterior estimates result for the models concerning legal and business characteristics of databases are presented in Figs. 6 and 7. Regarding the consent dimension, the lowest share of databases in which re-consent is required was estimated for electronic health records (68.5%; 95% CI 46.8%–85.0%), while the highest proportion for that outcome was estimated for genetic databases (79.9%; 95% CI 66.6%–89.8%). The highest estimates for the second consent outcome were observed for hospital information systems (HIS) (89.1%; 95% CI 71.2%–94.2%). The “no-consent” model for sharing anonymized patient health information in network electronic exchange for research purposes is the most common proportion estimated in health-related registries (12.7%; 95% CI 6.6%–21.1%). Furthermore, this model was estimated at the lowest value for hospital information systems (HIS) (7.2%; 95% CI 1.8%–18.7%).

Data ownership represented by patient awareness of data usage for further research, monitoring performance, service planning, audit, and quality assurance purposes was estimated to have the highest share in genetic databases (93.7%; 95% CI 84.3%–98.4%). Moreover, genetic databases were found to collect the highest proportion of genetically sensitive information (29.6%; 95% CI 22.0%–37.9%). The provision of mental health data as a sensitive information item is estimated to have the highest share in health-related registries (8.5%; 95% CI 5.0%–13.2%).

Legislative provisions concerning the primary and secondary use of data were estimated to have the highest share in the hospital information system (87.8%; 95% CI 74.9%–95.8%). The lowest share of legislative provisions was estimated for health-related registries (78.5%; 95% CI 68.4%–86.2%). Regarding data security, at least one national data security policy is observed in electronic medical records databases (90.8%; 95% CI 79.8%–97.1%). In addition, EMRs were estimated with the lowest value of immediate willingness to share data as a contribution to the goals of the Screen4Care project (14.1%; 95% CI 5.8%–27.8%). The highest reported proportion of data sharing dimension was found in the genetic database (18.7%; 95% CI 9.1%–32.0%).

Based on the posterior estimates, a ranking-based approach was used to determine the re-consent requirements, patients’ awareness their information may be used for further research and legislative provisions concerning the primary and secondary use of data order geographical scope for the responders of registry type databases and by the database type (Additional file 1: Table 4. Legal items – ranking by the posterior estimates of the Bayesian logit model according to geographical scope for the responders of registry-type databases and the database type). The highest ranking of the re-consent requirement was listed for international registries coordinated in Europe and European registries, containing health data of patients with neurological and neuromuscular disorders as well as metabolic and endocrine disorders. Patients included in international registries coordinated in Europe and European registries demonstrated the highest awareness about the use of their health information for further research, monitoring performance, service planning, audit, and quality assurance purposes. The existence of legislative provisions concerning the primary and secondary use of data was with the highest rank for European and regional Beta oxidation disorders and metabolic and endocrine disorders registries.

**Fig. 8:**
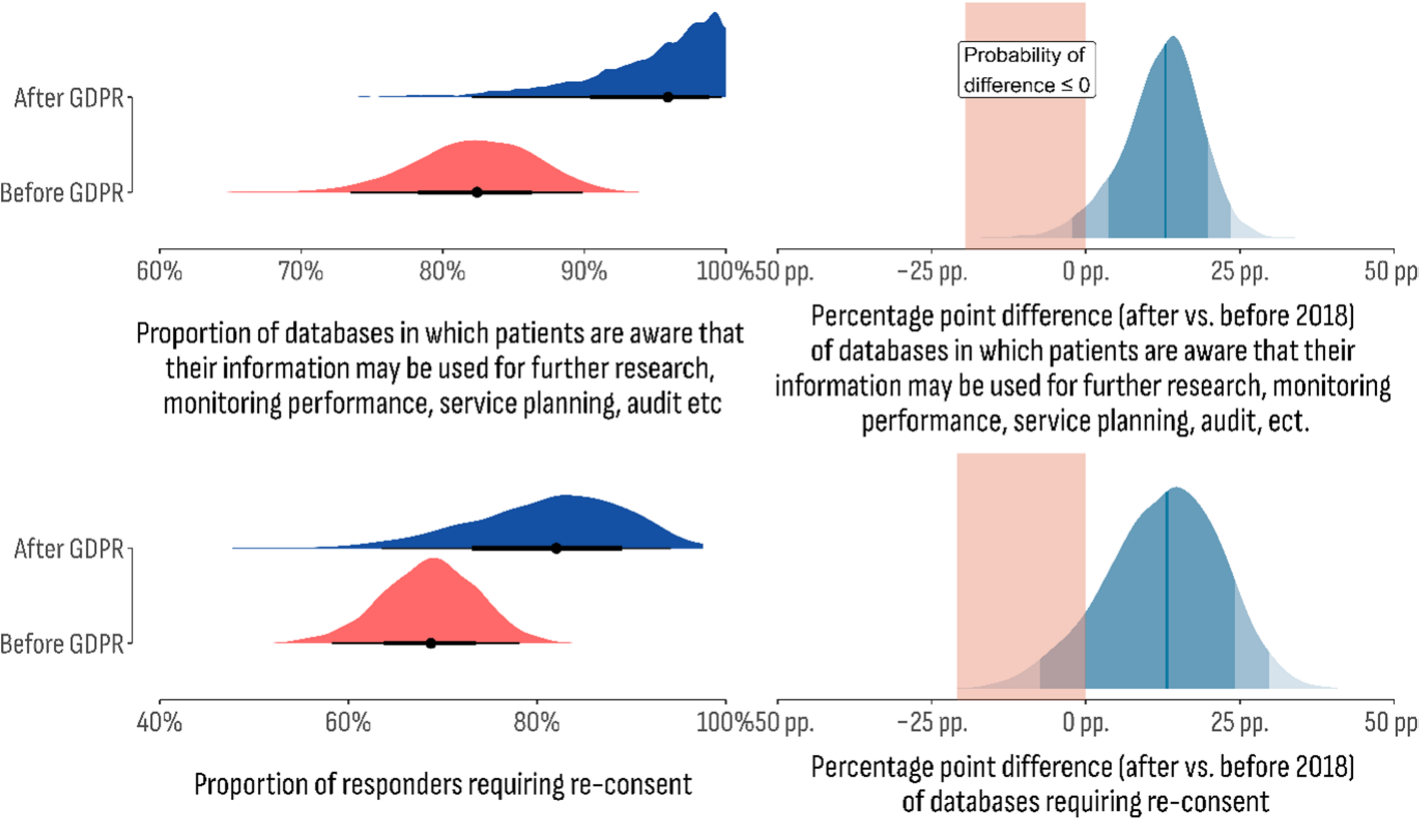
The Bayesian model fitted using GDPR implementation as the predictor variable and patients’ awareness of their information being used for further research, monitoring performance, service planning, audit, and quality assurance purposes as the response variable with a predefined outcome of interest

**Fig. 9:**
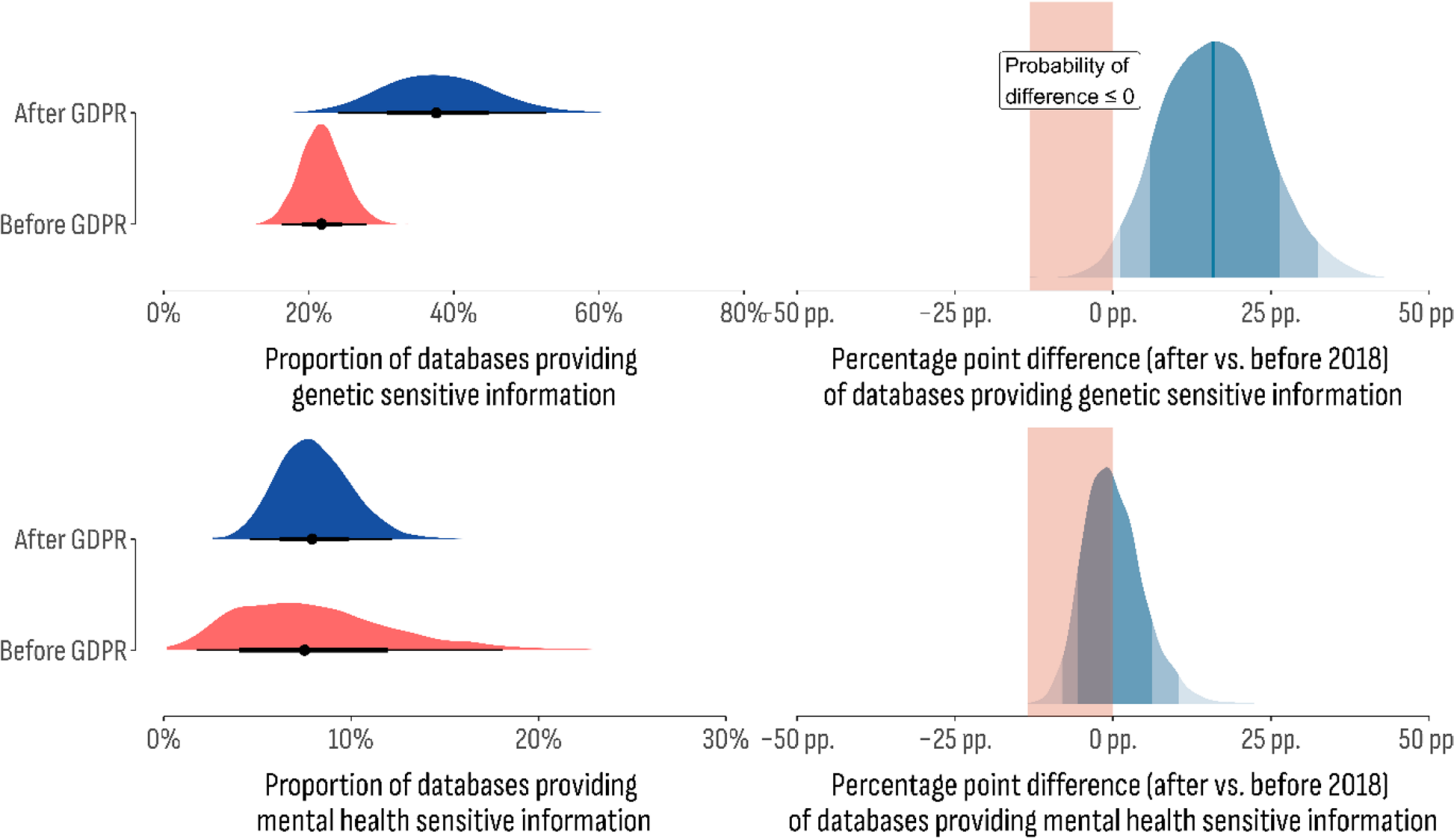
The Bayesian model fitted using GDPR implementation as the predictor variable and sensitive information as the response variable with a predefined outcome of interest

To estimate the effect of GDPR (General Data Protection Regulation) on database management characteristics, we compared the predefined outcome of interest in the consent, ownership, and sensitive data collection dimensions across databases established before and after the GDPR implementation (2018). One hot encoding was used to create a dummy variable based on the reported year of database establishment. Bayesian models then were fitted using the new variable as a predictor and the predefined outcome of interest as the response variable. The results are shown in Figs. 8 and 9.

Regarding data ownership, evaluated by the proportion of databases reporting patients’ awareness of their information being used for further research, monitoring performance, service planning, audit, and quality assurance purposes, the databases established before 2018 are estimated to have a lower posterior probability (80%; 95% CI 73.0%–89.46%) compared to those established after 2018 (95.8%; 95% CI 82.6%–99.7%). The median posterior difference between the two groups is 12.9 percentage points (95% CI: -1.0 p.p. to 23.7 p.p.). The GDPR effect on data ownership can be measured by the relative share of posterior draws suggesting a positive difference compared to those with a negative or null value. The latter is an estimate of the magnitude of the effect and is usually noted as the Bayes Factor (BF). In this case, the BF is 26.59, indicating that the data is 26.59 times more likely to favor the alternative hypothesis (proportion difference) compared to the null hypothesis (no difference). This suggests that the effect of GDPR on data ownership is significant.

The same approach was used for the consent dimension evaluated by the re-consent database requirement. Before GDPR implementation, the estimated positive outcome was 68.9% (95% CI 58.06%–78.3%), while after 2018, the estimated positive outcome was 81.9% (95% CI 62.6%–94.3%). The median posterior difference between the two groups is 12.6 percentage points (95% CI: -0.8 p.p. to 30.6 p.p.). The Bayes Factor is 8.54, indicating moderate evidence against the no-difference hypothesis.

Finally, sensitive data provision was compared between databases established before and after the GDPR regulations. Even though two outcomes—genetic and mental health data—were included in this dimension, a contracting relationship between them was observed. For instance, genetic data was found to be more likely to be provided after 2018 (37.6%; 95% CI 24.2%–53.5%), while the opposite was observed for mental health data (before GDPR: 8.0%; 95% CI 4.6%–12.3%; after GDPR: 7.5%; 95% CI 2.0%–17.8%). The estimated difference for genetic data (16.0 p.p., 95% CI 1.1 p.p.–32.9 p.p.) was associated with the corresponding BF of 59,6 indicating strong evidence in favor of alternative hypotheses and therefore a significant impact of GDPR enabling databases for genetic data exchange. However, the estimated values for mental health data provision indicated a decline compared to databases established after 2018. The observed difference (-0.4 p.p.; 95% CI -7.7 p.p. 10.3 p.p.) was associated with a BF of 1.6, indicating weak evidence in favor of the null hypothesis.

## Discussion

ML and AI are increasingly being used in healthcare and genetic research to improve patient outcomes and advance scientific knowledge. Further, their application can be beneficial for early diagnosis, improving prognosis, and treatment decisions in the field of RDs [24]. However, such technology relies heavily on the availability of high-quality data, which in the RD domain is often scarce and fragmented [25]. Moreover, the lack of standardized data sharing practices and interoperability standards across different domains can hinder the progress of these innovations [26].

### FAIRness recommendations

Assessing the FAIR principles that were developed to address these challenges [22] is intended to address specific recommendations and strengthen the process of AI and ML implementation to help RD patients. In the presented study, it was found that the overall adherence to FAIR principles varied significantly among the databases. A total of 25 respondents (17.9%) reported full compliance with all four FAIR components, indicating a significant portion of databases that have successfully implemented these principles. However, more than half of the respondents either could not provide an answer (*n* = 56, 40%) or indicated that the FAIR principles were not being applied on the site (*n* = 23, 16.4%). The latter, combined with the relatively low response rate (42.7%) for this section, suggests a lack of awareness and limited implementation of FAIR principles among the surveyed database stakeholders. This is consistent with other studies that have found low levels of FAIR compliance [27]. Therefore, the results underline the importance and need for targeted interventions to promote FAIR compliance and standardize data sharing practices across different domains. Such efforts are proven to be efficient and impactful in the RD and genetic research domains [28, 29].

In this research, a deeper exploration of each FAIR component aimed to identify specific strengths and weaknesses in the surveyed database management and sharing practices. A significant portion of respondents (51.7%) indicated that their data repository utilized a PID, a critical factor that enhances the discoverability and traceability of data [30]. Furthermore, 14 respondents (23.3%) reported multiple data releases with attached versions, which improves data tracking and version control [31]. These findings indicate that efforts have been made to ensure comprehensive access to datasets, fostering openness and transparency, which is proven to be an effective strategy for enhancing quality in RD registries [32]. However, only 45.9% of the respondents ensured accessibility either via a web browser or API, enabling data retrieval through standard protocols. This suggests that there is potential for enhancement in this aspect, specifically in clinical databases, where accessibility is comparatively lower. This can be a barrier to the development of database workflows needed for ML and AI technologies [33] Moreover, only 20 databases (33.9%) reported implementing data licensing, enabling reusability. This result emphasizes the considerable limitations of collaborative research and knowledge dissemination [34].

Database characteristics such as database type, diseases included in the data, and geographical scope of the database were also investigated as potential factors influencing FAIR adherence. Databases containing information on neuromuscular disorders and those with European scope demonstrated the highest overall FAIR adherence. Notably, genetic databases showed the highest proportion of positive responses to overall FAIR adherence, suggesting that these databases have made significant strides in adopting FAIR practices. This may be attributed to the emphasis on data sharing and standardization within the genetics research community [35]. In contrast, low FAIR assessment is found for databases of EHR and HIS. These clinical databases face unique challenges related to data privacy, security, and interoperability, which hinder their ability to fully implement the FAIR principles [36].

The higher FAIR compliance in databases focused on neuromuscular disorders could be attributed to the relatively specialized nature of these databases and patient advocacy which facilitated more focused and standardized data management practices [37, 38]. Additionally, recent initiatives specially designed for data on neuromuscular disorders might have contributed to the higher FAIR adherence in these databases [39, 40]. The influence of EU policies, efforts, and funding assistance that promote data sharing and FAIR implementation may be related to the observed FAIR adherence in databases with a European focus [41, 42]. European databases might also benefit from standardized data sharing frameworks and infrastructure, enabling smoother data exchange and collaboration across European countries [43].

Significant obstacle was observed for the reusability domain in the overall FAIR assessment. Our study revealed low levels of support for the FHIR and OMOP standards, with only 11.86% of respondents indicating adherence to the FHIR standard and 6.78% reporting compatibility with OMOP. These findings can be attributed to two primary factors. Firstly, metadata vocabularies remain highly technical and challenging for many stakeholders, especially within patient rare disease registries and those lacking sustainable funding for database maintenance and configuration [44, 45]. Additionally, it is evident that numerous data repositories have yet to adopt common metadata templates, despite the existence of such schemes tailored for rare diseases [23]. This underscores the pressing need for wider adoption and implementation of standardized metadata vocabularies to enhance data interoperability and facilitate broader reuse within the rare disease research community.As a result of this study several recommendations can be outlined. Adhering to these suggestions enables researchers to pinpoint databases that conform to FAIR principles, ensuring the availability of high-quality, readily accessible, and standardized data. These qualities are vital for effectively implementing ML and AI technologies in RD research. Utilizing such databases will lead to more precise and meaningful results, ultimately contributing to improved patient care and the advancement of scientific knowledge in this complex area of study.

1. Databases with standardized data sharing practices and data formats ensuring consistency and interoperability across different databases should be used as a primary data source for ML and AI applications.
2. When selecting databases for training datasets it is crucial to prioritize those that utilize persistent identifiers (PIDs). PIDs enhance the discoverability and traceability of data repositories ensuring consistent input for ML and AI algorithms.
3. To facilitate integration with ML and AI technologies databases should ensure data accessibility through web browsers or APIs. This allows for the retrieval and analysis of data.
4. Databases that offer multiple data releases with attached versions should be preferred, as data versioning enables improved data tracking and version control, which are vital for accurate ML and AI model training.
5. For ML and AI applications, it may be beneficial to consider specialized databases focused on RD domains like disorders. Such databases often provide standardized data suitable, for these applications. Researchers should actively pursue research projects to discover databases that follow practices, for data sharing and adhere to common protocols, for data exchange. This will greatly facilitate the integration of ML and AI.
6. Databases focused on specific RD domains, such as those for neuromuscular disorders, should be considered, as they may offer more comprehensive and standardized data suitable for ML and AI applications.
7. Collaborative research initiatives should be sought by researchers to identify databases with standardized data sharing practices and adherence to common data exchange protocols, facilitating ML and AI integration.
8. A thorough assessment of the database’s documentation should be conducted to ensure transparency and comprehensive information about data quality, format, and metadata, which are crucial for ML and AI model development.
9. European-scope databases, with their emphasis on data sharing and standardized practices, should be considered, as they may provide robust and FAIR data suitable for ML and AI research, particularly in the RD domain.
10. European databases might benefit from standardized data sharing frameworks and infrastructure for smoother data exchange and collaboration across European countries with high-quality, easily accessible, and standardized data provided.

## Legal and business recommendations

The GDPR provides enhanced protection for health care information in the EU, as reflected in the member countries implementing laws. The GDPR, which entered into force on 24 May 2016 and is applicable from 25 May 2018, creates a harmonized set of rules applicable to all personal data processing taking place in the EU [46]. National data protection authorities are responsible for monitoring and enforcing the application of the GDPR and other national data protection legislation that may be applicable in their territories. In our study, 75% of the respondents (with a proportion over 80% for HER and EMR) declared that there is at least one national data security policy regarding the technical standards to be used to ensure health data for primary use are processed and stored securely and 37.5% of them pointed out the existence of several. The results are similar to those reported in another European study on the topic [47]. Moreover, a regional health authority is traditionally primarily responsible for the containment of individual cases. Thus, it will depend on Member State legislation when in that chain data will be anonymized. Clarifications are however needed under which conditions the further processing of data in order to render them anonymous for the purpose of scientific research would be legitimate [48].

In our study, 76.8% of the respondents confirmed the presence of legislative provisions concerning the primary and secondary use of data. It can be particularly challenging to strike the correct balance between enabling good data use and protecting privacy when it comes to secondary use. Secondary use involves processing data for purposes other than those originally intended when information is gathered, and it may also involve data processors other than the primary data collectors, in contrast to primary use, where data are collected and then used for a specific purpose [47, 49–51]. In contrast with the study of Skovgaard et al., published in 2019, our results demonstrate that 83.9% of the respondents declare that patients are aware that their information may be used for further research, monitoring performance, service planning, audit, and quality assurance purposes etc. [52]. Moreover, awareness is of key importance for patients involved in RD research, and it could be argued that this becomes even more evident in data sharing, with the onus on researchers, institutions, and collaborations to recognize this as a responsibility. Rare disease patients’ perspectives are needed to contribute to the debate on the management, sharing and protection of data, in order to reconcile tensions within the research process with what matters most to patients [53]. There is also a risk of too much privacy protection in the RD context. Formal legal safeguards and strict transparency requirements leave organizations with less flexibility to share samples and data about RD patients, especially internationally, even where researchers seek explicit patient consent and/or patient involvement in data sharing governance [54].

The informed consent of the citizen is essential for data exchange [55]. The voluntary expression of consent is fundamental to ethical research practices. While patients with RDs often expect that data are shared for scientific advances, they are also concerned about being identified, a risk enhanced in the RD context [56]. In RD research, the consent processes have become increasingly complex, considering the current landscape of technological and genomic advances, together with the extensive collection and dissemination of data worldwide. This has been confirmed by the multiple components included in the consent process and authorization mechanisms for health records exchange in the various databases examined by us. In our study, the most commonly used consent models applied for sharing anonymized patient health information in network electronic exchange for research purposes are opt-in (16.2%) and opt-in with restrictions (10.8%). An additional challenge is the different types of collected consent, including consent for every use of data (30.4%), consent for broader categories of research (27.7%) and consent for all research (17.9%). The need for improving informed consent processes in international collaborative RD research is broadly discussed, namely, there is a need for effective consent in order to conduct effective research. To achieve this aim, the procedure shall address possible ethical and legal hurdles that could hamper research in the future, including opt-in, re-consent and opt-out strategies [57]. We consider this especially relevant while examining informed consent for RD research, in particular, when there are re-consenting requirements for data used in ways that do not fall in the original purpose of the respective registry, or other database research, which we found to be mandatory for 70.5% of the databases we collected responses from.

Although the GDPR harmonizes the regulations governing the processing of sensitive data, such as individual health information, Member States still have the option to establish legal grounds for processing health information. Furthermore, Article 9(4) clearly states that Member States are free to maintain or enact new restrictions, including requirements, in relation to the processing of genetic, biometric, or health data [46]. This could indicate that the GDPR would not be administered uniformly across all Member States in the domain of health. It may also imply that there may be disparities in how the GDPR is implemented within a single Member State, particularly where local law is in effect [48]. The findings of our survey show that 48.4% of the participants collect genetic data, and this is more likely to occur following GDPR's enforcement in 2018.

The responsible sharing of genetic and other health-related data shall be a foundational principle in data collection program management, including compliance with the obligations and norms set by international and national law and policies [49, 58]. According to the Framework for Responsible Sharing of Genomic and Health-Related Data [59], several core elements of responsible data sharing shall be respected, including transparency, data quality and security, privacy, data protection and confidentiality. The terms of data usage are a main quality element of a registry and by prioritizing ethical and legal standards, high quality registries can provide access to data on a platform that ensures data security and patient confidentiality [60]. A very small relative part of the participants (16.6%) declared willingness to share their database as a contribution to the goals of the Scree4Care EU project.

The EU is preparing governance frameworks that permit access to data in the near future. The aim is to increase trust in data intermediaries and boost data sharing inside the EU and between sectors in order to promote data availability and assist ethical and sustainable research and development processes [61].

The following recommendations could be given to facilitate the process of obtaining health information from various data sources for the development of ML algorithms for the screening and early detection of RDs:

1. Good practices as transparent data use and providing patients with information on how their data might be used for future research, performance monitoring, service planning, audit, and quality assurance purposes, among other things.
2. Precise legal grounds should be established for the data processing and provide special consideration to the use of informed consent.
3. Re-consenting requirements should be considered when selecting particular databases.
4. A solid understanding of data protection law should be obtained to guarantee that IT security standards are strictly followed.
5. If the results of data processing may benefit the identification of RD patients, pseudonymization of the data should be applied.
6. Researchers should be aware that data is collected in a manner that permits its utilization across systems without compromising their integrity and that it's readily available where needed.
7. Improved collaboration with ERNs and Healthcare institutions on country and EU level could accelerate EU and local initiatives to bring increased data sharing and accessibility for sustainable innovation in RDs diagnosis and treatment.
8. In order to effectively improve medical care for RD patients, additional efforts for aggregating information and expertise for healthcare professionals via projects and networks should be performed. The S4C project will further advance the overcoming of medical challenges with delayed diagnosis and treatment of RD patients through newborn genetic screening for RDs, such as neuromuscular disorders as one of multiple examples.

## Limitations

The outcomes of our research should be considered in terms of the limitations of our study design and sampling methods. This was a cross-sectional questionnaire survey that gives an illustration of the current context of health-related databases to respond to the need for rapid identification of RDs using ML technology. Thus, no changes in this environment could be examined over time. This is critical when discussing breakthrough fields such as ML, whose exponential development has already resulted in new EU legislation and initiatives for health-specific data sharing. The geographical scope of the study comprised EU and EEA (European Economic Area)-based health-related datasets, which may limit the generalizability of the results. Although the convenience sampling method was a relevant choice for the narrow and well-defined pool of respondents, the combination with the heterogeneity of the questionnaire might result in nonresponse bias. Given this disadvantage, the questionnaire design included an option for respondents to refer to experts regarding FAIR principles and legal and business information. To limit nonresponse bias, a questionnaire was sent out to 3032 individuals, 2212 of whom were ERN specialists, with the expectation that the most knowledgeable would fill out the survey and answer as many specific questions about the database as possible. Although many definitions and clarifications about organizational, FAIR and legal domains were provided, and the Screen4Care consortium aligned the questionnaire content and design, the survey concepts were complex and heterogeneous; thus, some respondents may not have fully understood the information included. Furthermore, it should be noted that different FAIR assessments tools may produce contrasting outcomes when applied to the same resource [62]. Therefore, it is crucial to interpret our findings in light of the specific aims and objectives of the S4C project, while also considering potential limitations in external validity. In addition, selection bias cannot be ruled out, as respondents may have been more informed about ML than nonparticipants.

## Conclusions

The technological innovation that brought digital transformation in healthcare – telehealth, ML, AI-enabled medical devices, blockchain electronic health records, automation, Internet of Things, Big data, etc. – demands large amounts of health data to be fed with. The most important results of our study demonstrate not enough sufficient FAIR principals’ adherence and low willingness of the EU health databases to share patient information, combined with some legislation incapacities, resulting in barriers to the secondary use of data. This landscape should be transformed in the near future by EU initiatives that already started as European Open Science Cloud (EOSC) and recently adopted EU Data Governance Act, followed by Personal Data Spaces (PDS) and the European Health Data Space (EHDS). These new EU governing structures are expected to build trust in data providers and stimulate data sharing to promote accessibility and support ethical and sustainable innovation in healthcare.

### Supplementary Information

**Supplementary Material 1.**

**Supplementary Material 2.**

**Supplementary Material 3.**

## Data Availability

The data that support the findings of this study are not openly available due to reasons of sensitivity and are available from the corresponding author upon reasonable request. Data are located in controlled access data storage at the Institute for Rare Diseases (Plovdiv, Bulgaria).

## Abbreviations

AI: Artificial intelligence
API: Application Programming Interface
CHERRIES: Checklist for Reporting Results of Internet E-Surveys
EEA: European Economic Area
EFPIA: European Federation of Pharmaceutical Industries and Associations
EHDS: European Health Data Space
EJP RD: European Joint Program on Rare Diseases
EMR: Electronic medical records
EOSC: European Open Science Cloud
EPIRARE: European Platform for Rare Disease Registries
ERN: European Reference Networks
ESS: Effective sample size
EU: European Union
FAIR: Findability, Accessibility, Interoperability, and Reusability
FHIR: Fast Healthcare Interoperability Resources
GDPR: General Data Protection Regulation
HER: Electronic health records
HIS: Hospital information system
HPO: Human phenotype ontology
ICD: International Classification of Diseases
IMI: Innovative Medicines Initiative
IQR: Interquartile range
IT: Information technology
ML: Machine learning
MRI: Magnetic resonance imaging
OMOP: Observational Medical Outcomes Partnership
PDS: Personal Data Spaces
PID: Persistent Unique Identifier
PROM: Premature rupture of membranes
RD: Rare disease
Screen4Care: Screen for Care

## Footnotes

1 National database refers to information collected and aggregated at the level of an entire country; regional database focuses on specific geographic areas within a country, such as states, provinces, or counties; local database pertains to information collected at the smallest geographic scale, such as cities, towns, or neighborhoods. National data offers a comprehensive view of a country as a whole, regional and local data provide increasingly granular insights into specific geographic areas, allowing for more targeted analysis and decision-making at different levels of healthcare governance and administration.

2 Federated metadata repository is a centralized system that aggregates and manages metadata from multiple distributed sources or systems while maintaining their autonomy; federated machine learning algorithms are a class of machine learning techniques designed to train models across multiple decentralized data sources while keeping the data localized and private.

3 European databases include the European reference networks and repositories of organized health-related information pertaining to Europe or specific European regions.

4 International databases are repositories of health-related information that encompass data from multiple countries or regions across the globe.
