# Supplementary material for: Landscape analysis of available European data sources amenable for machine learning and recommendations on usability for rare diseases screening
Source: Orphanet J Rare Dis. 2024 Apr 6;19:147. doi: 10.1186/s13023-024-03162-5 (PMC10998425; doi:10.1186/s13023-024-03162-5)
Supplement: Supplementary file 1 — Supplementary Material 1. [file 13023_2024_3162_MOESM1_ESM.pdf]

ADDITIONAL FILE 1

Table 1. Main rare disease groups and subgroup distribution.

	Responses (n)	Percent of Cases
**Metabolic and endocrine disorders**	**86**	**30.0%**
Gaucher disease Type I, Type II, Type III, GBA	32	39.5%
FABRY disease	43	53.1%
POMPE disease	32	39.5%
Familial isolated hypoparathyroidism due to impaired PTH secretion	21	25.9%
Multiple endocrine neoplasia type 1	33	40.7%
Alpha1-antitrypsin deficiency	21	25.9%
Primary hyperoxaluria	21	25.9%
Other	40	49.4%
Subgroup Total	*243*	*300.0%*
**Neurological and neuromuscular disorders**	**84**	**29.3%**
Proximal spinal muscular atrophy	36	45.6%
Duchenne muscular dystrophy	45	57.0%
Congenital myasthenia	33	41.8%
Familial amyloid polyneuropathy type I	20	25.3%
Autosomal recessive dopa- responsive dystonia	19	24.1%
Autosomal dominant dopa- responsive dystonia	19	24.1%
Limb-girdle muscular dystrophy	34	43.0%
Encephalopathy due to GLUT1 deficiency	23	29.1%
Other	36	45.6%
Subgroup Total	*265*	*335.4%*
**Hematological diseases**	**50**	**17.4%**
Haemophilia B	30	54.5%
Haemophilia A	30	54.5%

Factor XIII deficiency	24	43.6%
Factor VII deficiency	23	41.8%
Paroxysmal nocturnal haemoglobinuria	19	34.5%
Thrombotic thrombocytopenic purpura	27	49.1%
Other rare hematological diseases	39	70.9%
Subgroup Total	*192*	*349.1%*
**Beta oxidation disorders**	**27**	**9.4%**
Carnitine palmitoyl transferase deficiency type 2	17	70.8%
Defect of acyl CoA dehydrogenase very long chain fatty acids VLCHAD	16	66.7%
Defect of acyl CoA dehydrogenase long chain fatty acids LCHAD	19	79.2%
Carnitine deficiency secondary to medium-chain acyl-CoA dehydrogenase deficiency	17	70.8%
Other	7	29.2%
Subgroup Total	*76*	*316.7%*
**Other rare conditions**	**177**	**61.7%**
Cystic fibrosis	33	19.3%
X-linked adre0leukodystrophy	21	12.3%
0onan syndrome	41	24.0%
Osteogenesis imperfecta	37	21.6%
Leber hereditary optic neuropathy (LHON)	27	15.8%
Other	137	80.1%
Subgroup Total	*296*	*173.1%*
**I cannot answer**	**22**	**7.7%**
**Total of the main groups**	**446**	**155.4%**

* The overall main group and subgroups percentages exceed 100%, because these were a semi-closed enumerated questions.

Table 2. FAIR database characteristics

FAIR principles		n	%
	All FAIR principles are explicitly mentioned	25	17.9%
	FAIR principles are not applied on the site	23	16.4%
	I cannot answer	56	40.0%
	The F and A principles are explicitly mentioned	2	1.4%
	The FAIR principles are not explicitly mentioned, but there is a reference to sustainable and long-term storage.	34	24.4%
Data Service Units (data coordination center)			
	General research data policy is not supported by DCC	7	11.5%
	I cannot answer	19	31.2%
	There are dedicated staff members responsible for the implementation of the services	20	32.8%
	There is a dedicated central organizational unit (either virtual or physical) to provide the services needed to execute the policy. However, some conditions like very large data sets, or FAIR vocabularies are still not fully covered in the services.	8	13.1%
	We provide and/or facilitate all services for all aspects, also for domain-specific interoperability standards and storing large data.	7	11.5%
**Findability**			
Metadata persistency (DOI)			
	I cannot answer	12	20.0%
	No	10	16.7%
	Other	7	11.7%
	Yes	31	51.7%
Naming convention			
	I cannot answer	35	53.0%
	Typographical	17	25.8%

	Grammatical	10	15.2%
	Other	4	6.1%
Several data releases with versions attached			
	I cannot answer	11	18.33%
	No	35	58.33%
	Yes	14	23.33%
Metadata vocabularies			
	ORPHA code	26	27.96%
	International statistical classification of diseases and related health problems ICD	22	23.66%
	I cannot answer	20	21.51%
	other	13	13.98%
	snomed ct	7	7.53%
	medical subject headings MSH	4	4.30%
	dublin core	1	1.08%
**Accessibility**			
Availability option			
	All datasets	28	45.90%
	Data can’t be made available to third party	8	13.11%
	I cannot answer	1	1.64%
	Part of the database	24	39.34%
Data access software documentation			
	I cannot answer	20	33.33%
	No	25	41.67%
	Yes	15	25.00%
**Interoperability**			
Data is interoperable			
	I cannot answer	7	11.67%

	No	10	16.67%
	Yes	43	71.67%
FHIR standard supported			
	I cannot answer	36	61.02%
	No	16	27.12%
	Yes	7	11.86%
Medical outcomes partnership (OMOP)			
	I cannot answer	32	54.24%
	No	23	38.98%
	Yes	4	6.78%
**Re-usability**			
Data licenses permit re-usability			
	I cannot answer	28	47.46%
	Yes	20	33.90%
	No	11	18.64%
Data security			
	Encryption and pseudonymization	49	39.52%
	Identity and access management IDAM	27	21.77%
	Data loss prevention DLP	22	17.74%
	I cannot answer	8	6.45%
	Incident response plan	6	4.84%
	Other	6	4.84%
	Third party risk management	6	4.84%

Table 3. Overall characteristics of database management - legal and business information

Legal and business information		n	%
Legislative provisions concerning the primary and secondary use of data			
	I cannot answer	19	17.0%
	No	7	6.3%
	Yes	86	76.8%
There are national health data security policies regarding the technical standards to be used to ensure health data for primary use are processed and stored securely?			
	I cannot answer	26	23.2%
	There are no national data security policies	2	1.8%
	There are several national data security policies	42	37.5%
	There is one national data security policy	42	37.5%
Patients are aware that their information may be used for further research, monitoring performance, service planning, audit, quality assurance purposes, etc.			
	I cannot answer	10	8.9%
	No	8	7.1%
	Yes	94	83.9%
Required re-consent of patients when data is used in ways that do not fall within the original purpose of the registry			
	I cannot answer	8	7.1%
	No	25	22.3%
	Yes	79	70.5%
Type of consent collected			
	Consent for all research	20	17.9%
	Consent for broader categories of research on data	31	27.7%
	Consent for every use of data	34	30.4%
	I cannot answer	24	21.4%
	Refuse consent to all research	3	2.9%

Consent models applied for sharing anonymized patient health information in network electronic exchange for research purposes			
	I cannot answer	53	47.8%
	No consent	14	12.6%
	Opt-in	18	16.2%
	Opt-in with restrictions	12	10.8%
	Opt-out	10	9.0%
	Opt-out with exceptions	4	3.6%
Sensitive information provided			
	Genetics	61	48.4%
	I cannot answer	26	20.6%
	Mental health	19	15.1%
	Other	20	15.9%
Willing to share your database to contribute to the goals of the Scree4Care EU project?			
	I cannot take this decision	75	69.4%
	No, I won't share our database under no terms	15	13.9%
	Yes, I will share our database for free	9	8.3%
	Yes, I will share our database for potential cost	9	8.3%

Table 4. Legal items – ranking by the posterior estimates of the Bayesian logit model according to geographical scope for the responders of registry type databases and the database type

**Re-consent requirement**					
	Posterior mean	Posterior median	2.5 % CI	97.5% CI	Rank
Scope					
European registry	81.1%	82.4%	59.8%	95.2%	2
International registry (coordinated in Europe)	82.9%	86.6%	46.8%	99.5%	1
National registry	75.5%	76.0%	60.4%	87.7%	3
Regional registry	49.6%	49.8%	27.0%	72.4%	4
Disease type contained					
Beta oxidation disorders	54.2%	54.7%	26.3%	80.6%	4
Haematological diseases	47.5%	47.3%	26.2%	69.5%	5
Metabolic and endocrine disorders	75.8%	76.1%	61.4%	87.6%	2
Neurological and neuromuscular disorders	76.0%	76.5%	60.6%	88.4%	1
Other rare conditions	65.3%	65.4%	53.9%	75.7%	3
**Patients are aware that their information may be used for further research, monitoring performance, service planning, audit, and quality assurance purposes**					
	Posterior mean	Posterior median	2.5 % CI	97.5% CI	
Scope					
European registry	99.5%	100.0%	94.4%	100.0%	1
International registry (coordinated in Europe)	98.7%	100.0%	86.1%	100.0%	2
National registry	78.2%	78.9%	63.2%	89.4%	3

Other	46.2%	45.5%	1.6%	95.9%	5
Regional registry	74.3%	75.4%	51.3%	91.9%	4
Disease type contained					
Beta oxidation disorders	53.3%	53.6%	25.1%	79.5%	5
Haematological diseases	62.7%	63.1%	40.8%	81.5%	4
Metabolic and endocrine disorders	73.1%	73.5%	58.1%	85.8%	2
Neurological and neuromuscular disorders	69.6%	70.1%	53.0%	84.1%	3
Other rare conditions	86.1%	86.5%	77.2%	93.1%	1
**There are legislative provisions concerning the primary and secondary use of data**					
	Posterior mean	Posterior median	2.5 % CI	97.5% CI	
Scope					
European registry	87.1%	88.7%	67.3%	98.1%	1
International registry (coordinated in Europe)	65.8%	68.0%	27.4%	94.0%	4
National registry	78.3%	78.7%	64.1%	89.6%	3
Other	47.5%	45.9%	2.0%	96.7%	5
Regional registry	81.0%	82.3%	59.5%	95.7%	2
Disease type contained					
Beta oxidation disorders	82.0%	83.8%	56.1%	97.6%	1
Haematological diseases	73.4%	74.2%	51.8%	90.6%	4
Metabolic and endocrine disorders	81.1%	81.7%	67.6%	91.6%	2
Neurological and neuromuscular disorders	75.5%	76.0%	60.3%	88.3%	3
Other rare conditions	80.5%	80.9%	70.6%	88.5%	5
