# Supplementary material for: Landscape analysis of available European data sources amenable for machine learning and recommendations on usability for rare diseases screening
Source: Orphanet J Rare Dis. 2024 Apr 6;19:147. doi: 10.1186/s13023-024-03162-5 (PMC10998425; doi:10.1186/s13023-024-03162-5)
Supplement: Supplementary file 2 — Supplementary Material 2. [file 13023_2024_3162_MOESM2_ESM.pdf]

ADDITIONAL FILE 2

Table 1. Checklist for Reporting Results of Internet E-Surveys (CHERRIES) [53, 54]

Item category	Checklist item	Description
**Design**	Study design	In the period of March 2022 to December 2022, a cross-sectional study using a semi-structured questionnaire was conducted. A non-random convenience sampling method was used in recruiting the participants and a list of potential respondents was prepared, including all individuals that had been identified eligible to answer the questions – main contact person of a database that could be: health-related registry, electronic medical record (EMR), electronic health record (EHR), hospital information system (HIS), and repositories for genomics. Participants were recruited by means of individual emails. In addition, based on the heterogeneity of the questionary a non-random snowball sampling method was applied to target experts with extensive experience in FAIR principles for database management, organization, level of access and metadata or broad knowledge about legal, ethical, and business practices in data collection and operation with focus on consent and data ownership, sensitive information, data protection, legislation, data sharing and fees.
**Ethics**	Ethics approval	The anonymous nature of the survey did not require ethics committee approval. The study was conducted according to ethical guidelines established by the Declaration of Helsinki.
	Informed consent	The questionnaire introductory panel started with a general description of "Screen4Care" project, outlined the aim of collecting the particular information on the topic of interest and ended with a consent statement for the use of the anonymous data, which was agreed to before filling out the survey questions by the respondent. The survey participation was entirely voluntary. The respondents were informed that it would take approximately 25 minutes

		to complete the form, that all responses were confidential and anonymous, and that reporting would be on an aggregate level only. Consent was indicated when respondents clicking the “I agree to participate” option on starting page.
	Data protection	To maintain data security, specialized survey software (LimeSurvey platform, Enterprise plan version) and local servers were employed. No personal information was linked to survey findings. The fully anonymized dataset is stored on password-protected PCs.
**Development and pre-testing**	Development and testing	The design of the self-completed questionnaire survey instrument was based on information drawn from relevant scientific publications, quantitative and qualitative research, and scoping review on challenges in mapping European rare disease databases, relevant for ML-based screening technologies in terms of organizational, FAIR and legal principles (13). Usability had been discussed within Screen4Care Consortium for one month and technical functionality of the electronic questionnaire was tested by members in our team before fielding the questionnaire.
**Recruitment process and description of the sample having access to the questionnaire**	Open survey versus closed survey	This was a closed survey. A CSV files of different type of databases were prepared and e-mail of contact person/s included. Then the file was uploaded in LimeSurvey platform, duplicates were automatically omitted. There were total of four rounds of Questionnaire virtual dissemination.
	Contact mode	Limited initial contacts with the potential participants were made. ERN coordinators were contacted and informed about the survey with the kind request to ask their members actively to participate in this research.
	Advertising the survey	The survey was not advertised.

**Survey administration**	Web / E-mail	This was a web-based survey, with respondents directed to the LimeSurvey website. The online survey platform automatically collected responses, which were then stored on secure local servers.
	Context	LimeSurvey is a web-based statistical survey program that is free and open source. It is web server-based software that allows users to build and publish online surveys, collect replies, generate statistics, and export the resultant data to other applications via a web interface.
	Mandatory/voluntary	The survey participation was entirely voluntary. Potential respondents were selected based on eligible criteria. A non-random convenience sampling method was used in recruiting the participants and in addition, based on the heterogeneity of the questionary a non-random snowball sampling method was applied.
	Incentives	No incentives were offered.
	Time/Date	Responses were collected between March to December 2022. Four rounds were executed within the timeframe of the study.
	Randomization of items or questionnaires	Neither randomization nor alternation of items or questionnaires was applied.
	Adaptive questioning	Adaptive questioning techniques were employed. Based on previous responses, relevant survey items were displayed.
	Number of Items	A dynamic number of questions were available per page based on the adaptive nature of the questionnaire. Thus, although the full survey included a total of 81 items, not all respondents answered all of them.

	Number of screens (pages)	The entire survey was spread across 8 screens (49 pages of PDF file).
	Completeness check	All survey items were considered mandatory, and respondents were prompted to fill out any remaining items before exiting the survey page that featured the item. A nonresponse option such as "I cannot answer" was included in 31 items; both "I cannot answer" and "Other" with text answer were listed in 12 items; and "Other" without text answer was option in 5 items.
	Review step	Respondents were no longer able to change their responses once submitted the questionnaire. Meanwhile they could stop working on survey, they could do back and forth changing answers – LimeSurvey saves the responses and allows changes any time before the final submission.
**Response rates**	Unique site visitor	3032 contacts identified.
	View rate (Ratio of unique survey visitors/unique site visitors)	32.5% - 986 unique survey visitors / 3032 contacts identified
	Participation rate (Ratio of unique visitors who agreed to participate/unique first survey page	75.6% - 746 unique visitors agreed to participate /986 unique first survey page visitors.

	visitors)	
	Completion rate (Ratio of users who finished the survey/users who agreed to participate)	44.23% - 330 users who finished the survey satisfying the analysis requirements/ 746 unique visitors who agreed to participate
**Preventing multiple entries from the same individual**	Cookies used	No
	IP Check	IP was automatically checked for token generation process and ensuring databases - responder match. IP addresses were not collected by researchers.
	Log file analysis	Not used
	Registration	Entry to the survey was via a unique login provided to each invitee to the survey
**Analysis**	Handling of incomplete questionnaires	Only completed questionnaires were included in the final dataset
	Questionnaires submitted with an atypical timestamp	No respondents were removed from the survey for completing the items too quickly
	Statistical correction	Bayesian analysis was implemented as a preferred method for small sample sizes and subgroup analysis.
