# Supplementary material for: Landscape analysis of available European data sources amenable for machine learning and recommendations on usability for rare diseases screening
Source: Orphanet J Rare Dis. 2024 Apr 6;19:147. doi: 10.1186/s13023-024-03162-5 (PMC10998425; doi:10.1186/s13023-024-03162-5)
Supplement: Supplementary file 3 — Supplementary Material 3. [file 13023_2024_3162_MOESM3_ESM.pdf]

ADDITIONAL FILE 3

Table 1. Structure and content of the questionnaire survey instrument

**PANELS**	**NUMBER AND TYPES OF QUESTIONS**	**CONTENT**
Introduction	**1** (1)[a] (0)[b] (0)[c] (0)[d] (0)[e] (0)[f]	conscent for participation in the study;
Administrative	**2** (1)[a] (0)[b] (0)[c] (0)[d] (0)[e] (1)[f]	number of databeses responsible for; name/s of the other database/s;
Screening	**26** (2)[a] (3)[b] (3)[c] (6)[d] (0)[e] (12)[f]	type of data your organization is operating with; year of database establishment and activity - observational period, last time of new data collection, data update frequency, number of active cases/patients, new cases during the last year of observation; geographical scope; type of the registry; total number of observations/cases; clinical or diagnostic data included; type of data included; date of patient death registered; email address of another contact person informed about specificities of the data; rare diseases groups included; rare hematological diseases included; other rare hematological diseases; Beta oxidation disorders included; other Beta oxidation disorders; neurological and neuromuscular disorders included; other neurological and neuromuscular disorders; metabolic and endocrine disorders included; other metabolic and endocrine disorders; rare conditions included; other rare conditions;
FAIR-ness	**28** (17)[a] (1)[b] (3)[c] (0)[d] (0)[e] (7)[f]	short info and option to continue or quit if lack of expertise; FAIR principles mentioned in the database policy; general research data policy supported by dedicated service units; FAIR data inclusion as a criterion in the initial and continuous evaluation process; database data discoverable with metadata, identifiable and locatable by means of a standard identification mechanism; naming conventions; number of releases data and specific

		versions attachments; data availability type; consideration of local only analysis; reason the data non-sharing option; data access committee availability; methods or software tools to access data; documentation about the software needed to access the data included in the metadata repository; well described conditions for accessing the data; manner of ascertaining the identity of the person accessing the data; data and metadata vocabularies, standards or methodologies followed to ensure data interoperability; data exchange and re-use between different parties; FHIR standard adoption to support eSource data exchange; Observational Medical Outcomes Partnership Common Data Model implementation; type of software for database management systems; licensed data for re-use; data usage by third parties; provisions in place for data security; informed consent for data sharing and long term preservation included in questionnaires dealing with personal data; national/funder/sectorial/departmental procedures for data management – name them; interfaces for querying existence; email address of another contact person informed about specificities of FAIR-ness;
Legal and Business	**23** (13)[a] (0)[b] (2)[c] (0)[d] (2)[e] (6)[f]	short info and option to continue or quit if lack of expertise; legislative provisions concerning the primary and secondary use of data; national health data security policies regarding the technical standards to be used to ensure health data for primary use are processed and stored securely; patients' awareness that their information may be used for further research, monitoring performance, service planning, audit, and quality assurance purposes etc.; re-consent requested when data is used in ways that do not fall within the original purpose of the registry; form of the collected consent; models applied to capture and maintain patient consent electronically; type of consent

		collected; components are included in the consent process; system identification and definition of the roles and responsibilities of important actors (“controllers”, “processors” and “subjects”); system definition of the consent management mechanisms required for further processing of data; level of readiness and capability of the system to adapt to different privacy policies; data removal enabled; interoperability between systems enabled; consent models applied for sharing anonymized patient health information in network electronic exchange for research purposes; categories of sensitive information provided; different considerations for providing health information; legal rules and ethics procedures imposed in relation to data delivery and data usage; type of costs for managing the registries and other data collection programs; willingness to share your database to contribute to the goals of Scree4Care EU project; Is a privacy impact assessment tool available; email address of another contact person informed about specificities of Legal and Business practices;
End of survey	**1** (0)[a] (0)[b] (0)[c] (0)[d] (0)[e] (1)[f]	additional comments on the survey.

[a] closed-ended single choice questions

[b] semi-closed selective questions with a text answer

[c] semi-closed enumerated question with a text answer

[d] semi-closed enumerated question without a text answer

[e] matrix of questions

[f] open-ended questions
